# Effects of enzyme-treated soy oligopeptide on intestinal health, feed preference, and growth performance in nursery pigs

**DOI:** 10.5713/ab.25.0153

**Published:** 2025-06-10

**Authors:** Lan Zheng, Jung Yeol Sung, Sung Woo Kim

**Affiliations:** 1 Department of Animal Science, North Carolina State University, Raleigh, NC, USA

**Keywords:** Enzyme-treated Soy Oligopeptide, Feed Preference, Growth, Intestinal Health, Pig, Soybean Meal

## Abstract

**Objective:**

The objective was to evaluate the effects of replacing soybean meal (SBM) with enzyme-treated soy oligopeptide (ESO) in nursery pig diets on intestinal health, growth performance, and feed preference.

**Methods:**

In Exp. 1, 128 pigs (average 5.2 kg) were housed in pens (4 pigs/pen), assigned to 4 diets supplemented with 0%, 1%, 2%, or 3% of ESO, and fed for 32 d. On d 32, blood, tissues, and mucosa from the duodenum and jejunum were collected. In Exp. 2, 24 pigs (average 6.2 kg) were assigned to 6 pens (4 pigs/pen). Each pen had two separate feeders containing two diets with 0% or 2% of ESO for 27 d.

**Results:**

Increasing dietary ESO tended to quadratically change serum tumor necrosis factor-α (p = 0.059; minimum at 1.6% of ESO) and duodenal villus height to crypt depth ratio (p = 0.062; maximum at 1.8% of ESO). Increasing dietary ESO linearly reduced feed intake both linearly and quadratically (p<0.05) during phase 2 (maximum at 0.9% of ESO). Increasing dietary ESO both linearly and quadratically changed (p<0.05) weight gain during phase 2 (maximum at 1.0% of ESO). Increasing dietary ESO linearly changed (p<0.05) and tended to quadratically change (p = 0.077) weight gain during the overall phase. Increasing dietary ESO tended to linearly change (p = 0.059) gain to feed ratio during phase 1 and quadratically change (p<0.05) gain to feed ratio during phase 2 (maximum at 1.4% of ESO). Feed preference of the diet containing 2% of ESO was negatively associated with post-weaning days (R^2^ = 0.542).

**Conclusion:**

Gradually replacing SBM with ESO at levels from 1.4% to 1.8% enhanced intestinal health and gain to feed ratio, whereas exceeding 0.9% to 1.0% reduced feed intake and weight gain, suggesting that optimal level of ESO in nursery pig diets is 0.9%.

## INTRODUCTION

Soybean co-products such as soybean meal (SBM) are widely used protein supplements that provide amino acids in pig diets. However, soybean contains allergenic proteins that are among the most detrimental anti-nutritional compounds for pigs [1]. Typical examples of allergenic proteins are glycinin and β-conglycinin in pigs, which are the main storage protein in soybean [2]. However, these proteins can also lead to inflammation in the intestinal tract when pigs consume high quantities of soybean co-products due to their allergenic proteins [3]. Particularly, weaned pigs are hypersensitive to allergenic proteins, which may exacerbate post-weaning stress. Therefore, pig producers should be cautious not to supplement excessively high concentrations of soybean co-products in nursery diets [4].

Various processing methods have been used to address the issue related to allergenic proteins in soybean by using microbial fermentation, enzymatic hydrolysis, or chemical treatment [1]. Processed soy proteins (e.g., soy protein concentration, fermented SBM, and enzyme-treated SBM meal) with small peptides and reduced allergenicity have been well used in feeding nursery pigs for their beneficial aspects with high nutrient digestibility and efficiency of amino acid utilization [5–8] and with improved intestinal health in relation to balanced mucosal microbiota, attenuated inflammatory response, reduced oxidative damages, and improved villus functions [9–11]. Another example of processed soy proteins is soy protein hydrolysate, which is produced by hydrolyzing soybeans with enzymes, and enzyme-treated soy oligopeptide (ESO) is a typical example of soy protein hydrolysate [12]. In addition to the benefit of reduced allergenic protein concentrations, ESO contains various bioactive compounds, such as isoflavone and saponin, which potentially improve intestinal health of pigs [13].

Our previous studies showed that increasing the inclusion rate of processed soy proteins in nursery diets (e.g. soy protein concentrate, fermented SBM, and enzyme-treated SBM) improved intestinal health and feed efficiency, but excessive inclusion reduced appetite for unclear reasons [6,10,11]. Considering the results of previous studies on processed soy proteins, this study aimed to test the hypothesis that partially replacing SBM with ESO enhances intestinal health and feed efficiency in nursery pigs. The background for the hypothesis is that ESO contains a lower amount of allergenic protein compared with SBM but the dose level should be controlled due to its impact on reduced appetite. To test this hypothesis, the objective was to evaluate the effects of gradually replacing SBM with ESO in nursery pig diets on intestinal health, growth performance, and feed preference. Based on the results of this study, the optimal level of ESO in diets was determined to improve intestinal health and growth performance in nursery pigs.

## MATERIALS AND METHODS

### Molecular weight distribution of peptides in enzyme-treated soy oligopeptide

The ESO was treated with mixture of enzymes to hydrolyze allergenic proteins. The molecular weight distribution of peptides in ESO was assessed using an analytical ultracentrifuge (ProteomeLab XL-A; Beckman Coulter) following the procedure outlined by Schuck et al [14]. Homogenized samples were centrifuged at 12,300×g for 15 min at 4°C using an Eppendorf MiniSpin (Eppendorf). A total of 200 scans were collected at 6-min intervals. Proteins were prepared with 2 mL of 20 mM Tris-HCl buffer containing 0.1% sodium dodecyl sulfate and 5 mM dithiothreitol at pH 7.4. Aliquots (110 μL) of the sample solution were loaded into 6-sector sample cells. Absorbance was monitored at 280 nm for the loaded samples. Sedimentation velocity data were analyzed using the SEDFIT software (version 11.8; National Institutes of Health) to generate the sedimentation coefficient distribution for the protein samples (Table 1).

### Composition of amino acids and allergenic proteins in enzyme-treated soy oligopeptide

The concentrations of amino acids in ESO (Table 2) were determined by ion-exchange chromatography with post-column derivatization with ninhydrin, following the procedure described by Jang et al [15]. Before analysis, samples were liberated from the protein by hydrolysis with 6 N HCl for 24 h at 110°C. Methionine and cysteine were analyzed as methionine sulfone and cysteic acid after cold performic acid oxidation overnight before hydrolysis. Tryptophan was determined after NaOH hydrolysis for 22 h at 110°C. The concentrations of glycinin and β-conglycinin in ESO were measured using Glycinin ELISA Kit (BA-UBT002; Unibiotest) and β-Conglycinin ELISA Kit (BA-UBT001; Unibiotest), following the procedure described by Deng et al [16]. Briefly, proteins were extracted using the sample extractant provided in the kits, then shaken vigorously for 16 h at 25°C, centrifuged at 4,000×g, and diluted 70-fold with the sample diluent. Absorbance was measured at 450 and 630 nm, respectively, and protein concentrations were calculated based on a standard curve generated from known standard concentrations and their corresponding absorbance values.

### Experiment 1

#### Animals, experimental design, and experimental diets

Hundred-twenty-eight weaned pigs (64 barrows and 64 gilts) with an initial average weight of 5.2±0.5 kg were randomly allocated to four dietary treatments in a randomized complete block design with initial weight and sex as blocks. Each treatment had 8 pens. Four pigs (2 barrows and 2 gilts) were housed in pens (1.2 m×1.2 m) equipped with a feeder and a nipple drinker, allowing them free access to both feed and water. The four diets included corn-SBM-based diets supplemented with 0%, 1%, 2%, or 3% of ESO at the expense of SBM (Table 3). Pigs were fed for 32 d (phase 1: d 0 to 11 and phase 2: d 11 to 32). Each diet was formulated to meet or exceed the nutrient requirements for nursery pigs provided by NRC [17].

#### Sample and data collection

Weight of pigs and feed disappearance of each pen were measured on d 0, 11, and 32 to determine average daily feed intake (ADFI), average daily gain (ADG), and gain to feed ratio. On d 32, blood samples were collected from the jugular vein of each using BD Vacutainers and then centrifuged at 3,000×g for 15 min at 4°C to obtain serum. After collecting blood, the pig with median weight per pen was euthanized using a captive bolt followed by exsanguination to collect 20 cm of mid-duodenal and mid-jejunal tissues. The mucosa was removed from the first 15 cm of the collected duodenum and jejunum, then quickly frozen in liquid nitrogen and stored at −80°C. The remaining 5 cm was fixed in a 10% buffered formalin solution.

#### Immune responses

One gram of the collected duodenal and jejunal mucosa was homogenized with a homogenizer (Tissuemiser; Thermo Fisher Scientific) to assess levels of immunoglobulin A (IgA), immunoglobulin G (IgG), tumor necrosis factor (TNF)-α, and interleukin (IL)-10. After homogenization, the mixture was centrifuged at 14,000×g for 30 min at 4°C to collect the supernatant. The total protein content of the supernatant was determined using the Pierce BCA Protein Assay kit (23225#; Thermo Fisher Scientific). The resulting total protein concentration was used to standardize the immune response measurements. The ELISA kits for pig IgA (E101–102) and pig IgG (E101–104) from Bethyl Laboratories were used to analyze duodenal and jejunal mucosa. Additionally, TNF-α and IL-10 levels in duodenal and jejunal mucosa were measured using ELISA kits from R&D Systems. Serum was also analyzed for IgG, TNF-α, and IL-10 using the same methods used for mucosa.

#### Intestinal morphology in the duodenum and jejunum

Duodenal and jejunal tissue samples were embedded in paraffin and 5 μm thick sections were prepared using a rotary microtome. The sections were stained with hematoxylin and eosin. Measurements of villus height, villus width, and crypt depth in the duodenum and jejunum were taken at 40 × magnification, as described by Deng et al [11]. For each slide, the lengths of 10 well-oriented intact villi and their associated crypts were recorded. To assess the percentage of Ki-67 antigen-positive cells in the duodenum and jejunum, immunohistochemical staining was conducted on the histological sections [18]. The intact crypts were isolated, and Image J software was utilized to calculate the ratio of Ki-67 positive cells to total cells in the jejunal crypt. Crypt cell proliferation was calculated as follows:


(1) Crypt cell proliferation, %=(Ki-67 postive cells/Total cells)×100%.

#### Tight junction proteins in the duodenum and jejunum

Two samples of duodenal and jejunal tissues of pigs fed each diet was measured tight junction proteins as described by Jang et al [19]. Duodenal and jejunum tissue samples (50 mg) were homogenized in RIPA lysis with a protease inhibitor, then centrifuged at 10,000×g at 4°C for 10 min to collect the supernatant. Protein concentration was adjusted to 2 μg/μL and denatured before SDS-PAGE. After electrophoresis, proteins were transferred to a polyvinylidene difuoride membrane membrane, blocked with 5% skim milk, and incubated overnight with primary antibodies claudin (Sigma-Aldrich) diluted 1:400, occludin (Abcam) diluted 1:100, zonula occludens-1 (Santa Cruz Biotechnology) diluted 1:150, and β-actin (Cell Signaling Technology). Following incubation with horseradish-conjugated secondary antibodies, bands were detected using a chemiluminescent method (ECL; Amersham). Band densities were analyzed with image software (LI-COR Biosciences), and relative densities were calculated by normalizing to β-actin.

### Experiment 2

#### Feed preference test

Twenty-four weaned pigs (12 barrows and 12 gilts) with an initial average weight of 6.2±0.8 kg were randomly assigned to six pens in a randomized complete block design with initial weight and sex as blocks and four pigs (2 barrows and 2 gilts) per pen. Each treatment had 6 pens. Each pen (1.2 m×1.2 m) was equipped with a nipple drinker and two separate feeders containing different diets. The two experimental diets were corn-SBM-based diets without or with 2% of ESO (Table 4). The position of the two feeders in each pen was switched every 3 d to minimize positional preference. Pigs had unlimited access to feed and water. Feed disappearance of each pen was recorded every 3 d from d 0 to 27.

### Statistical analysis

The MIXED procedure in SAS (SAS Institute) was used for Exp. 1, with dietary treatment as a fixed effect and initial weight and sex as random effects. Orthogonal polynomial contrasts were used to determine linear and quadratic effects of increasing dietary ESO. The quadratic effects of ESO on the parameters were investigated by the RSREG procedure of SAS to find the level of ESO reaching the minimum or maximum value of the parameter. In Exp. 2, the REG procedure of SAS was used to regress feed preference for the diet containing 2% ESO to the diet without ESO on post-weaning days. A pig was the experimental unit for immune responses and morphology in the duodenum and jejunum in Exp. 1. A pen was the experimental unit in immune responses in serum and growth performance data in Exp. 1 and data in Exp. 2. Significance and tendency were determined at p<0.05 and 0.05≤p<0.10, respectively.

## RESULTS

**Figure 1:**
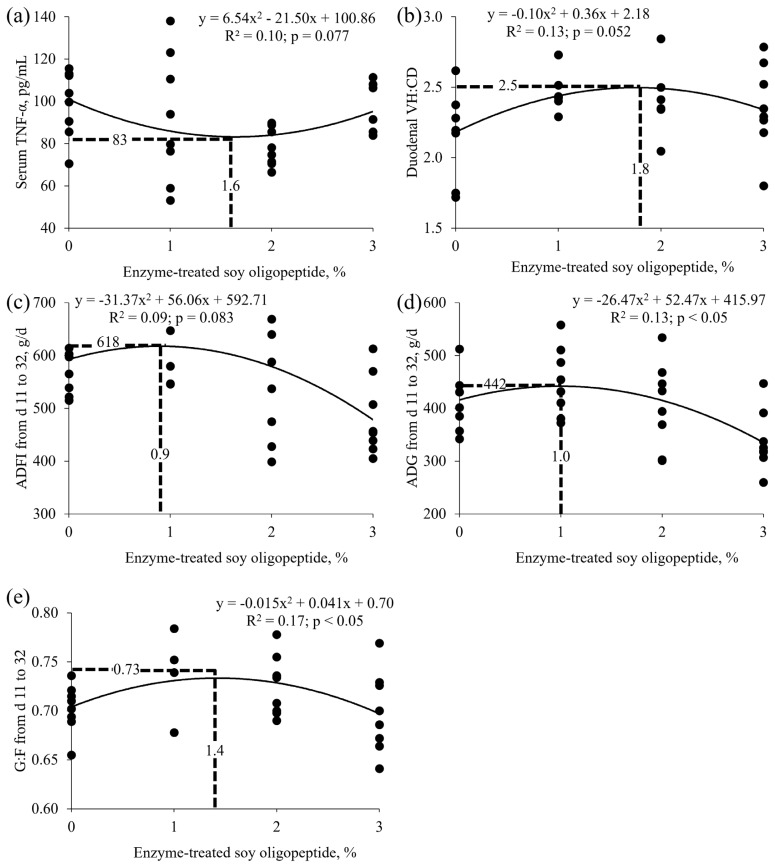
The enzyme-treated soy oligopeptide level for reaching the minimum or maximum value of parameters. (a) Serum tumor necrosis factor (TNF)-α level of nursery pigs fed diets varying enzyme-treated soy oligopeptide. The enzyme-treated soy oligopeptide level for reaching the minimum value is 1.6%. (b) Duodenal villus height to crypt depth (VH:CD) of nursery pigs fed diets with increasing levels of enzyme-treated soy oligopeptide. The enzyme-treated soy oligopeptide level for reaching the maximum value is 1.8%. (c) Average daily feed intake (ADFI) of nursery pigs fed diets with increasing levels of enzyme-treated soy oligopeptide during d 11 to 32. The enzyme-treated soy oligopeptide level for reaching the maximum value is 0.9%. (d) Average daily gain (ADG) of nursery pigs fed diets with increasing levels of enzyme-treated soy oligopeptide during d 11 to 32. The enzyme-treated soy oligopeptide level for reaching the maximum value is 1.0%. (e) Gain to feed ratio (G:F) of nursery pigs fed diets with increasing levels of enzyme-treated soy oligopeptide during d 11 to 32. The enzyme-treated soy oligopeptide level for reaching the maximum value is 1.4%.

The molecular weight distribution of peptides in ESO includes two fractions (32,650 Da or 863 Da), with an average molecular weight of 4,709 Da (Table 1). The concentrations of glycinin and β-conglycinin were lower in ESO compared with those in SBM (Table 2). Increasing dietary ESO did not affect IgA, IgG, and IL-10 in serum and mucosa in the duodenum and jejunum but it tended to quadratically change (p = 0.059) TNF-α in serum (Table 5). Increasing dietary ESO did not affect tight junction proteins in the duodenum and jejunum (Table 6). Increasing dietary ESO tended to quadratically change villus height to crypt depth ratio in the duodenum (p = 0.062; Table 7). Increasing dietary ESO changed feed intake both linearly and quadratically (p<0.05) during phase 2 and linearly (p<0.05) during the overall phase (Table 8). Increasing dietary ESO both linearly and quadratically changed (p<0.05) weight gain during phase 2. Increasing dietary ESO linearly changed (p<0.05) and tended to quadratically change (p = 0.077) weight gain during the overall phase. Increasing dietary ESO tended to linearly change (p = 0.059) gain to feed ratio during phase 1 and quadratically change (p<0.05) gain to feed ratio during phase 2. The optimal levels for reaching the minimum value of TNF-α in serum and maximizing the value of villus height to crypt depth ratio in the duodenum was 1.6% and 1.8%, respectively (Figure 1). The optimal levels for reaching the maximum values of ADFI, ADG, and gain to feed ratio during d 11 to 32 were 0.9%, 1.0%, and 1.4%, respectively (Figure 1).

**Figure 2:**
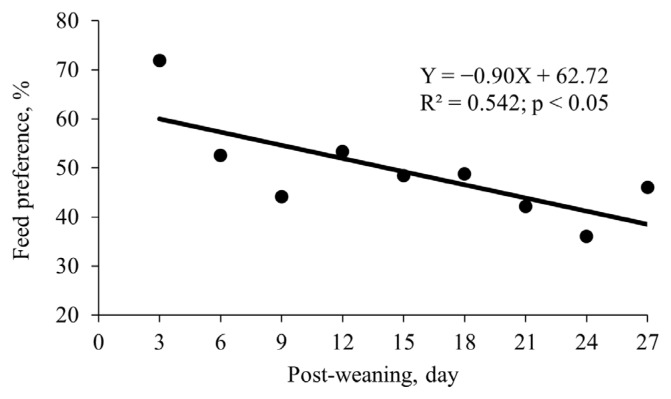
Relationship between post-weaning day and feed preference for the diet containing 2.0% enzyme-treated soy oligopeptide to the diet without enzyme-treated soy oligopeptide when fed to nursery pigs (Exp. 2). Each pen was equipped with two separate feeders containing two diets without or with 2% of enzyme-treated soy oligopeptide.

Feed preference was greater for the diet containing 2% of ESO from d 0 to 3, whereas lower from d 21 to 24 compared with the diet without ESO (p<0.05; Table 9). Feed preference of the diet containing 2% of ESO linearly decreased with increasing post-weaning days (R^2^ = 0.542; Figure 2).

## DISCUSSION

Glycinin and β-conglycinin have distinct molecular weight distributions of peptides. Glycinin has a molecular weight ranging from 300,000 to 380,000 Da, with its acidic and basic subunits weighing 35,000 Da and 20,000 Da, respectively [20]. In contrast, β-conglycinin has a molecular weight between 150,000 and 200,000 Da, with the α, α′, and β subunits weighing 72,000 Da, 68,000 Da, and 52,000 Da, respectively [21]. The greatest molecular weight fraction of peptides in ESO used in this study (32,650 Da and 12.1% of peptidues) overlaps with acidic subunits of glycinin (35,000 Da). However, the allergenic proteins in ESO would have been efficiently hydrolyzed during processing because of its low proportion and average molecular weight distribution (4,709 Da), as supported by the very low concentrations of glycinin and β-conglycinin in ESO. Therefore, processing soybean with enzymes to produce ESO may potentially increase its nutritional value.

In Exp. 1, the impact of a gradual increase in dietary ESO (0%, 1%, 2%, or 3%) was investigated on intestinal health and growth performance of nursery pigs. Tumor necrosis factor-α is a pro-inflammatory cytokine that promotes inflammation [19]. The reduction of TNF-α in serum up to 1.6% of ESO indicates alleviation of systemic inflammation [22], which is partly attributed to the decrease in dietary allergenic proteins by replacing SBM with ESO. The increase in serum TNF-α beyond 1.6% of ESO may be attributed to decreased ADFI, which could reduce the supply of energy and nutrients necessary to support the immune system of pigs [23]. Specifically, pigs are often exposed to various subclinical challenges including pathogens and heat stress [24]. When energy and nutrient intake falls short of physiological demands, as may have occurred in pigs fed 3% of ESO, the resulting deficit may trigger systemic inflammation [25,26]. Furthermore, the change in serum TNF-α due to dietary ESO is unlikely to be caused by intestinal inflammation because no signs of intestinal inflammation were observed in this study [19]. Dietary ESO may have indirectly caused systemic inflammation by affecting tissues other than the duodenum and jejunum, but the mechanisms underlying this remain unclear. The quadratic change in the villus height to crypt depth ratio in the duodenum (maximum at 1.8% of ESO) suggests an improvement in nutrient absorptive capacity due to ESO, primarily resulting from a reduction in hypersensitivity and oxidative stress caused by allergenic proteins [27,28]. The increase in the gain to feed ratio from 0% to 1.4% of ESO during phase 2 (d 11 to 32) can be partly attributed to the quadratic change of the serum TNF-α and villus height to crypt depth ratio in the duodenum. However, an attention is needed when applying the optimal levels for ESO suggested in this study when formulating diets due to high variation and a low coefficient of determination in estimating the optimal levels.

In Exp. 1, ADFI during phase 1 was not affected by dietary ESO. However, ADFI increased when pigs were fed up to 0.9% of ESO, but it decreased when pigs were fed beyond 0.9% of ESO during phase 2. The ADG during phase 2 followed a similar pattern to that of ADFI, starting to decrease when pigs were fed 1.0% of ESO. This decrease in ADG is primarily attributed to the reduction in ADFI because dietary ESO did not affect immune responses (IgA, IgG, and IL-10), and tight junction proteins, intestinal morphology (villus height/width, crypt depth, and Ki-67) in the duodenum and jejunum except for duodenal villus height to crypt depth ratio. Similar to this study, increasing the inclusion rate of processed soy proteins (e.g., soy protein concentration, fermented SBM, and enzyme-treated SBM meal) in diets improved intestinal health and gain to feed ratio up to a threshold level, but decreased feed intake, and consequently, weight gain of nursery pigs beyond the threshold [6,10,11]. The reason for non-significant difference in intestinal health parameters by dietary ESO remains unclear but it may be partially attributable to the presences of animal protein supplements, particularly for blood plasma. Blood plasma is not a simple source of amino acids, but it also contains bioactive compounds such immunoglobulins and albumin as to enhance intestinal health status of pigs [29,30]. Therefore, the presence of blood plasma might have masked the beneficial effects of replacing SBM with ESO. However, this speculation does not fully account for the changes in serum TNF-α and the villus height to crypt depth ratio in the duodenum.

The reduced appetite due to processed soy proteins can be speculated as follows: The first speculation is related to the amino acid profiles and molecular weight of processed soy proteins. The concentration of hydrophobic amino acids (e.g., phenylalanine and leucine), which are responsible for suppressing appetite, is usually high in processed soy proteins [5,12,31,32]. The appetite-suppressant properties of processed soy proteins may be enhanced by the release of hydrophobic amino acid side chains that were previously concealed before hydrolysis through enzymatic digestion or fermentation [33]. However, this speculation may not be applicable to ESO used in this study because the concentrations of leucine and phenylalanine were lower in ESO compared with SBM. Moreover, the degree of appetite suppression may depend on a specific range of molecular weights of processed soy proteins [34]. For example, Cho et al [35] reported that appetite suppression of peptides in different soy protein hydrolysates was the greatest with molecular weights between 2,000 and 4,000 Da, whereas it was the lowest for peptides under 1,000 Da. In contrast, peptides with molecular weights below 1,000 Da were the most appetite-suppressant in several soy protein hydrolysates [36]. Considering the discrepancy, the molecular weight of processed soy proteins may not directly reflect the degree of appetite suppression because the conditions of hydrolysis of anti-nutritional compounds and the nutritional composition of soybeans may vary. Another possibility is that processed soy proteins may promote the secretion of anorectic hormones in enteroendocrine cells (e.g., cholecystokinin, glucagon-like peptide 1, etc.), which reduce appetite of pigs [16,37]. The anorectic effects of processed soy proteins might be related to their bioactive components (e.g., isoflavones). However, it is debatable which component is responsible for reduction in feed intake, and the mechanisms behind it remain unclear [38–40].

Based on the results of Exp. 1, the ADFI of pigs was maximum at 0.9% of ESO, but it decreased when pigs were fed beyond 0.9% of ESO during phase 2. To validate reduced appetite of pigs by ESO in Exp. 1, feed preference test for the diets without or with 2% of ESO was conducted in Exp. 2. Although ADFI started to decline beyond 0.9% of ESO in Exp. 1, 2% of ESO was used in Exp. 2 to ensure a pronounced reduction in appetite. Additionally, it was worthwhile to test the impact of 2% of ESO on preference because the optimal dietary ESO level for the other response criteria was up to 1.8%. The greater feed preference for the diet containing 2% ESO from d 0 to 3 contradicts the observation that feed preference for the diet containing 2% ESO was not greater and was even lower compared with the diet without ESO after d 3. It remains unclear why pigs consumed more of the diet containing 2% of ESO compared with diet without ESO from d 0 to 3. It is speculated that receptors for appetite suppression by ESO in very young pigs may not have developed, which requires experimental validation. However, although the consumption of the diet containing 2% of ESO was greater compared with the diet without ESO during d 0 to 3, the actual difference between the two diets was very low because feed intake of nursery pigs is typically very low immediately after weaning [11]. Feed preference for the diet containing 2% ESO was negatively associated with post-weaning days, indicating that the appetite-suppressant property of ESO became more pronounced as pigs grew. Therefore, the result on ADFI in Exp. 1 is corroborated by the result of the feed preference test in Exp. 2.

## CONCLUSION

Gradually replacing SBM with ESO at levels from 1.4% to 1.8% optimized intestinal health and gain to feed ratio in nursery pigs. However, exceeding 0.9% to 1.0% reduced feed intake and weight gain, suggesting that optimal level of ESO in nursery pig diets is 0.9%.

## Figures and Tables

**Table 1:** Molecular weight distribution of enzyme-treated soy oligopeptide measured by analytical ultracentrifuge

Molecular weight (Da)	Percentage (%)	Average molecular weight (Da)^1)^
32,650	12.1	4,709
863	87.9	

1) The average molecular weight was calculated as the weighted sum of the molecular weights of the enzyme-treated soy oligopeptide based on their respective percentages.

**Table 2:** Composition of amino acids and allergnic proteins in soybean meal and enzyme-treated soy oligopeptide (as-is basis)

Item	Soybean meal^1),2)^	Enzyme-treated soy oligopeptide
Arginine (%)	3.45	3.91
Histidine (%)	1.28	1.08
Isoleucine (%)	2.14	1.47
Leucine (%)	3.62	2.36
Lysine (%)	2.96	2.82
Methionine+cysteine (%)	1.36	1.57
Phenylalanine (%)	2.40	1.91
Threonine (%)	1.86	1.24
Tryptophan (%)	0.66	0.56
Valine (%)	2.23	1.72
Glycinin (mg/g)	113	6
β-conglycinin (mg/g)	125	3

1) The composition of amino acids in soybean meal was adapted from NRC [17].

2) The composition of glycinin and β-conglycinin in soybean meal was adapted from Deng et al [16].

**Table 3:** Feedstuff composition of diets used in Exp. 1

ESO (%)	Phase 1 (d 0 to 11 post-weaning)				Phase 2 (d 11 to 32 post-weaning)			
	
	0.0	1.0	2.0	3.0	0.0	1.0	2.0	3.0
Feedstuff (%)								
Corn	43.13	43.20	43.29	43.36	54.45	54.50	54.56	54.67
Soybean meal	20.00	19.00	18.00	17.00	23.50	22.50	21.50	20.50
Whey permeate	20.00	20.00	20.00	20.00	10.00	10.00	10.00	10.00
Poultry meal	5.00	5.00	5.00	5.00	5.00	5.00	5.00	5.00
Blood plasma	4.00	4.00	4.00	4.00	2.30	2.30	2.30	2.30
Fish meal	4.50	4.50	4.50	4.50	-	-	-	-
ESO	-	1.00	2.00	3.00	-	1.00	2.00	3.00
Poultry fat	1.30	1.30	1.25	1.25	1.80	1.80	1.80	1.75
L-Lysine HCl	0.47	0.47	0.47	0.46	0.49	0.49	0.49	0.49
DL-Methionine	0.21	0.20	0.20	0.20	0.19	0.19	0.18	0.18
L-Threonine	0.15	0.15	0.16	0.16	0.15	0.15	0.15	0.16
L-Tryptophan	0.01	0.01	0.01	0.01	-	-	-	-
L-Valine	0.03	0.03	0.04	0.04	0.04	0.04	0.04	0.04
Limestone	0.75	0.71	0.65	0.62	0.87	0.82	0.77	0.75
Dicalcium phosphate	0.05	0.03	0.03	-	0.81	0.81	0.81	0.76
Vitamin premix^1)^	0.03	0.03	0.03	0.03	0.03	0.03	0.03	0.03
Mineral premix^2)^	0.15	0.15	0.15	0.15	0.15	0.15	0.15	0.15
Salt	0.22	0.22	0.22	0.22	0.22	0.22	0.22	0.22
Total	100.00	100.00	100.00	100.00	100.00	100.00	100.00	100.00
Calculated nutrient composition (as-fed basis)								
ME (kcal/kg)	3,402	3,404	3,404	3,406	3,400	3,402	3,404	3,404
Crude protein (%)	23.5	23.5	23.5	23.5	21.7	21.7	21.7	21.7
SID Lys (%)	1.50	1.50	1.50	1.50	1.35	1.35	1.35	1.35
SID Met+Cys (%)	0.82	0.82	0.82	0.82	0.74	0.74	0.74	0.74
SID Thr (%)	0.88	0.88	0.88	0.88	0.79	0.79	0.79	0.79
SID Trp (%)	0.25	0.25	0.25	0.25	0.22	0.22	0.22	0.22
SID Val (%)	0.95	0.95	0.95	0.95	0.86	0.86	0.86	0.86
Calcium (%)	0.85	0.85	0.85	0.85	0.80	0.80	0.80	0.80
STTD phosphorus (%)	0.45	0.45	0.45	0.45	0.40	0.40	0.40	0.40

1) The vitamin premix provided the following per kilogram diet: 3,968 IU of vitamin A; 1,190 IU of vitamin D_3_; 20 IU of vitamin E; 0.012 mg of vitamin B_12_; 4.0 mg of riboflavin; 33 mg of niacin; 6.6 mg of d-pantothenic acid; 1.2 mg of menadione; 0.012 IU of biotin.

2) The mineral premix provided the following per kilogram diet: 17 mg of Cu; 0.297 mg of I; 110 mg of Fe; 33 mg of Mn; 0.297 mg of Se; 110 mg of Zn.

ESO, enzyme-treated soy oligopeptide; ME, metabolizable energy; SID, standardized ileal digestible; STTD, standardized total tract digestible.

**Table 4:** Feedstuff composition of diets used in Exp. 2

ESO (%)	Phase 1 (d 0 to 11 post-weaning)		Phase 2 (d 11 to 27 post-weaning)	
	
	0.0	2.0	0.0	2.0
Feedstuff (%)				
Corn	43.13	43.29	54.45	54.56
Soybean meal	20.00	18.00	23.50	21.50
Whey permeate	20.00	20.00	10.00	10.00
Poultry meal	5.00	5.00	5.00	5.00
Blood plasma	4.00	4.00	2.30	2.30
Fish meal	4.50	4.50	-	-
ESO	-	2.00	-	2.00
Poultry fat	1.30	1.25	1.80	1.80
L-Lysine HCl	0.47	0.47	0.49	0.49
DL-Methionine	0.21	0.20	0.19	0.18
L-Threonine	0.15	0.16	0.15	0.15
L-Tryptophan	0.01	0.01	-	-
L-Valine	0.03	0.04	0.04	0.04
Limestone	0.75	0.65	0.87	0.77
Dicalcium phosphate	0.05	0.03	0.81	0.81
Vitamin premix^1)^	0.03	0.03	0.03	0.03
Mineral premix^2)^	0.15	0.15	0.15	0.15
Salt	0.22	0.22	0.22	0.22
Total	100.00	100.00	100.00	100.00
Calculated nutrient composition (as-fed basis)				
ME (kcal/kg)	3,402	3,404	3,400	3,404
Crude protein (%)	23.5	23.5	21.7	21.7
SID Lys (%)	1.50	1.50	1.35	1.35
SID Met+Cys (%)	0.82	0.82	0.74	0.74
SID Thr (%)	0.88	0.88	0.79	0.79
SID Trp (%)	0.25	0.25	0.22	0.22
SID Val (%)	0.95	0.95	0.86	0.86
Calcium (%)	0.85	0.85	0.8	0.8
STTD Phosphorus (%)	0.45	0.45	0.4	0.4

1) The vitamin premix provided the following per kilogram diet: 3,968 IU of vitamin A; 1,190 IU of vitamin D_3_; 20 IU of vitamin E; 0.012 mg of vitamin B_12_; 4.0 mg of riboflavin; 33 mg of niacin; 6.6 mg of d-pantothenic acid; 1.2 mg of menadione; 0.012 IU of biotin.

2) The mineral premix provided the following per kilogram diet: 17 mg of Cu; 0.297 mg of I; 110 mg of Fe; 33 mg of Mn; 0.297 mg of Se; 110 mg of Zn.

ESO, enzyme-treated soy oligopeptide; ME, metabolizable energy; SID, standardized ileal digestible; STTD, standardized total tract digestible.

**Table 5:** Systemic and intestinal immune responses of pigs fed diets with increasing supplementation levels of enzyme-treated soy oligopeptide (Exp. 1)

Item	Enzyme-treated soy oligopeptide (%)				SEM	p-value	
	
	0.0	1.0	2.0	3.0		Linear	Quadratic
IgA							
Duodenum (μg/mg protein)	3.45	2.93	3.56	3.14	0.60	0.899	0.920
Jejunum (μg/mg protein)	1.12	1.26	1.13	1.19	0.16	0.927	0.819
IgG							
Serum (mg/mL)	1.08	1.08	1.02	1.16	0.11	0.701	0.529
Duodenum (μg/mg protein)	1.09	1.21	1.40	1.35	0.13	0.105	0.525
Jejunum (μg/mg protein)	0.71	0.77	0.77	0.89	0.13	0.258	0.737
TNF-α							
Serum (pg/mL)	98.9	91.7	78.2	98.5	7.3	0.652	0.059
Duodenum (pg/mg protein)	0.73	0.75	1.43	0.83	0.28	0.271	0.126
Jejunum (pg/mg protein)	0.47	0.95	0.77	0.73	0.33	0.652	0.372
IL-10							
Serum (pg/mL)	71.4	72.8	71.9	67.4	4.1	0.441	0.425
Duodenum (pg/mg protein)	2.95	2.95	3.25	2.97	0.27	0.721	0.553
Jejunum (pg/mg protein)	2.59	3.09	2.66	2.75	0.27	0.966	0.238

Each least squares mean represents eight observations.

IgA, immunoglobulin A; IgG, immunoglobulin G; TNF-α, tumor necrosis factor-α; IL-10, interleukin-10.

**Table 6:** Tight junction proteins in the duodenum and jejunum of pigs fed diets with increasing supplementation levels of enzyme-treated soy oligopeptide (Exp. 1)

Item	Enzyme-treated soy oligopeptide (%)				SEM	p-value	
	
	0.0	1.0	2.0	3.0		Linear	Quadratic
Duodenum							
Claudin	0.50	0.40	0.39	0.41	0.07	0.388	0.436
Occludin	0.18	0.19	0.20	0.20	0.03	0.793	0.908
Zonula occludens-1	0.32	0.33	0.35	0.37	0.02	0.108	0.730
Jejunum							
Claudin	0.87	0.86	0.83	0.80	0.03	0.123	0.774
Occludin	0.18	0.18	0.19	0.18	0.03	0.920	0.953
Zonula occludens-1	0.26	0.22	0.19	0.21	0.09	0.703	0.723

Each least squares mean represents two observations.

Relative band density represents each tight junction’s band ratio compared with β-action’s band density.

**Table 7:** Intestinal morphology of pigs fed diets with increasing supplementation levels of enzyme-treated soy oligopeptide (Exp. 1)

Item	Enzyme-treated soy oligopeptide (%)				SEM	p-value	
	
	0.0	1.0	2.0	3.0		Linear	Quadratic
Duodenum							
Villus height (μm)	514	549	512	525	24	0.978	0.666
Villus width (μm)	100	94	103	93	7	0.471	0.579
Crypt depth (μm)	241	221	214	223	11	0.174	0.135
VH:CD	2.2	2.5	2.4	2.4	0.1	0.252	0.062
Ki-67 (%)	20.2	21.7	20.4	19.7	1.3	0.647	0.415
Jejunum							
Villus height (μm)	437	449	449	424	17	0.612	0.295
Villus width (μm)	83	81	82	78	2	0.150	0.407
Crypt depth (μm)	186	182	176	182	13	0.586	0.449
VH:CD	2.4	2.5	2.5	2.4	0.2	0.835	0.250
Ki-67 (%)	22.1	22.7	21.1	21.4	1.2	0.276	0.873

Each least squares mean represents eight observations.

VH:CD, villus height to crypt depth ratio.

**Table 8:** Growth performance of pigs fed experimental diets with increasing supplementation levels of enzyme-treated soy oligopeptide (Exp. 1)

Item	Enzyme-treated soy oligopeptide (%)				SEM	p-value	
	
	0.0	1.0	2.0	3.0		Linear	Quadratic
Body weight (kg)							
d 0	5.2	5.2	5.2	5.2	0.3	0.700	0.450
d 11	7.1	6.9	7.1	6.7	0.4	0.210	0.611
d 32	15.8	16.4	15.2	13.8	0.8	0.013	0.108
ADFI (g/d)							
Phase 1 (d 0 to 11)	227	239	214	216	27	0.304	0.654
Phase 2 (d 11 to 32)	588	636	564	484	51	0.008	0.046
Overall (d 0 to 32)	463	480	444	392	43	0.019	0.140
ADG (g/d)							
Phase 1 (d 0 to 11)	171	153	164	136	23	0.128	0.706
Phase 2 (d 11 to 32)	413	451	406	338	31	0.009	0.019
Overall (d 0 to 32)	336	352	316	273	25	0.004	0.077
Gain to feed ratio							
Phase 1 (d 0 to 11)	0.741	0.644	0.707	0.629	0.050	0.059	0.708
Phase 2 (d 11 to 32)	0.703	0.739	0.725	0.698	0.014	0.638	0.036
Overall (d 0 to 32)	0.727	0.731	0.720	0.696	0.029	0.278	0.484

Each least squares mean represents eight observations.

ADFI, average daily feed intake; ADG, average daily gain.

**Table 9:** Feed preference based on 3-day feed intake (%) of nursery pigs (Exp. 2)

Item	Enzyme-treated soy oligopeptide (%)		SEM	p-value

	0.0	2.0		
Feed preference (%)				
d 0 to 3	28.1	71.9	7.3	<0.001
d 3 to 6	47.4	52.6	5.1	0.628
d 6 to 9	55.9	44.1	5.0	0.262
d 9 to 12	46.7	53.3	2.9	0.275
d 12 to 15	51.6	48.4	5.7	0.795
d 15 to 18	51.2	48.8	5.5	0.837
d 18 to 21	57.8	42.2	6.6	0.253
d 21 to 24	63.9	36.1	7.3	0.048
d 24 to 27	54.0	46.0	8.6	0.662

Each least squares mean represents six observations. Each pen was equipped with two separate feeders containing two diets without or with 2% of enzyme-treated soy oligopeptide.
